# Ligand Fishing: A Remarkable Strategy for Discovering Bioactive Compounds from Complex Mixture of Natural Products

**DOI:** 10.3390/molecules21111516

**Published:** 2016-11-11

**Authors:** Rongjie Zhuo, Hao Liu, Ningning Liu, Yi Wang

**Affiliations:** 1 Faculty of Materials Science and Chemical Engineering, Ningbo University, Ningbo 315211, China; 2 College of Pharmaceutical Sciences, Zhejiang University, Hangzhou 310058, China; liuhao922@zju.edu.cn; 3 TCM Research Center, Tianjin University of Traditional Chinese Medicine, Tianjin 300193, China; 18867141533@163.com

**Keywords:** affinity chromatography, Traditional Chinese Medicine, screening active compounds

## Abstract

Identification of active compounds from natural products is a critical and challenging task in drug discovery pipelines. Besides commonly used bio-guided screening approaches, affinity selection strategy coupled with liquid chromatography or mass spectrometry, known as ligand fishing, has been gaining increasing interest from researchers. In this review, we summarized this emerging strategy and categorized those methods as off-line or on-line mode according to their features. The separation principles of ligand fishing were introduced based on distinct analytical techniques, including biochromatography, capillary electrophoresis, ultrafiltration, equilibrium dialysis, microdialysis, and magnetic beads. The applications of ligand fishing approaches in the discovery of lead compounds were reviewed. Most of ligand fishing methods display specificity, high efficiency, and require less sample pretreatment, which makes them especially suitable for screening active compounds from complex mixtures of natural products. We also summarized the applications of ligand fishing in the modernization of Traditional Chinese Medicine (TCM), and propose some perspectives of this remarkable technique.

## 1. Introduction

Natural products have played an indispensable role in healthcare systems throughout history, providing a huge and invaluable resource for the discovery of novel drug candidates. Traditional Chinese medicines (TCMs), widely used in Eastern Asian countries, have been regarded as an important use of natural products for the therapy of various diseases [1]. The discovery of bioactive constituents from TCMs that contribute to their well-known therapeutic effects is critical in the modernization of TCM. In the past two decades, tremendous efforts have been made for the identification of active components from TCMs, which is highlighted by artemisinin (qinghaosu) with its antimalarial effect as the most successful and impressive example and a gift from TCM to the world [2]. However, due to the chemical complexity of TCMs and other natural products, methods with satisfied sensitivity and efficiency for lead discovery are still in demand.

The typical identification process procedures often began with chemical separation followed by dereplication and biological assays. Numerous inactive compounds are also isolated along with active compounds during repeated isolation and purification steps, which make the process extremely time-consuming, labor intensive and poorly efficient. In line with accelerating advances of bio-guided techniques, affinity selection coupled with chromatography has attracted great attention because of its superiority in rapid screening [3]. Affinity-based screening assay, depending on the principle of macromolecular target-ligand binding, has been considered as one of the most convenient and efficient methods to separate potential ligands from complex mixture. These affinity-based screening assays can be applied to investigate multiple interacting pairs involved in biological systems, including antigen-antibody, receptor-ligand, enzyme-inhibitor/activator, and protein-protein interactions [4]. Thus, various types of targets can be utilized for affinity-based assays, including enzymes, receptors, neurotransmitters, transport proteins, DNA and any other bio-macromolecules, even cell membranes and living cells.

Bioanalytical screening techniques have been proposed as advanced alternatives to the classical bioassay-guided fractionation for active compound identification [5,6,7]. Taking advantage of diverse target immobilization methods and quick development of state-of-art analytical approaches, ligand fishing has emerged as a convenient and efficient technique to fish out ligands from complex mixtures. It was applied to screen potential ligand from crude tissue extracts and cell homogenates initially [8,9,10], and currently employed to fish out potential ligands from mixtures of natural products. In ligand fishing experiments, any compound with an affinity to the immobilized target will be retained for further analysis while non-binding compounds will remain in the extract and be discarded. Van Breemen, from University of Illinois at Chicago, has contributed a series of studies on affinity ultrafiltration mass spectrum since the middle of 1990s, establishing on-line ultrafiltration mass spectrometry for identifying drug metabolites formed by hepatic cytochromes P450 [11]. The high-selectivity and high-throughput of ligand fishing assays is achieved by specific enrichment of active components of various affinity selection techniques used in the system, which include, but are not limited to, centrifugation, ultrafiltration, equilibrium dialysis, microdialysis, magnetic beads, and affinity chromatography. The following analysis step is generally performed by analytical instruments such as HPLC or MS.

## 2. Classification of Ligand Fishing Strategies

**Figure 1:**
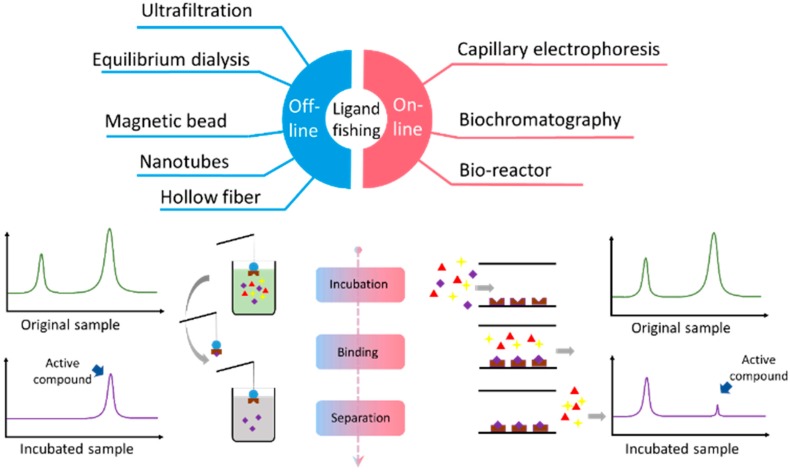
Scheme of ligand fishing applied to screening active compounds from complex mixtures. The left part shows the off-line mode: the affinity selection step usually performed independently, and the binding complexes were removed to eluent to obtain the active compounds, then using LC or MS to analyze them. The right part shows the on-line mode: the target molecules were immobilized inside of the chromatographic system, and the analysis and separation were simultaneous. The signal of active compounds would be lower than the original sample because of the affinity binding.

Depending on whether the separation and analysis are performed at the same time, ligand fishing approaches can be classified into on-line and off-line mode (as shown in Figure 1). During the off-line mode, active compounds trapped by immobilized bio-molecules such as enzymes or receptors on the stationary phase need to be first washed off than subjected to analysis. For on-line mode, the incubated sample solution is directly analyzed by chromatographic approaches after the bioextraction phrase and active ingredients from the mixture can be tentatively determined by comparison with chromatograms of the original sample.

### 2.1. Commonly Used Techniques in Off-Line Mode Ligand Fishing

In off-line mode ligand fishing, affinity selection and analysis are two independent steps. It is more versatile to combine various techniques to meet the different needs of natural products. In addition, less pretreatment of samples would be required for affinity separation. The first part is the bio-active extract separation through affinity selection, and various solid phases are applied. After fishing out the active compounds, it will turn to the analysis and identification by HPLC or MS. The ligand-bound complex can be separated from the unbound compounds via ultrafiltration, dialysis, affinity purification, size-exclusion chromatography, etc. Besides, many approaches based on bio-guided are also widely applied. Here, detailed discussions are provided

#### 2.1.1. Ultrafiltration

As a solution-phase screening technology, affinity ultrafiltration separates effective compounds from complex mixtures based on their highly specific affinity interactions to a particular target on the filter membrane. While the mixture through the ultrafiltration membrane, the specifically selected ligands retained on the filter membrane, separated by centrifugal force, were subsequently released from the complex after treatment with an appropriate eluent, then they were analyzed by liquid chromatography or mass spectrometry.

Nowadays, affinity ultrafiltration has been widely used in screening active compounds from traditional Chinese medicines. For example, Li et al. developed an effective method of ultrafiltration liquid chromatography with photodiode array detection coupled to electrospray ionization tandem mass spectrometry (UF-LC-MS) to screen and identify anti-α-glucosidase compounds from hawthorn leaf flavonoids extracts [12]. Yang et al. presented a new method based on UF-HPLC-DAD-MS to screen and identify inhibitors of tyrosinase from *Mulberry leaves* [13]. Song et al. develop a novel strategy based on ultrafiltration LC-MS and in silico molecular docking to screen inhibitors of xanthine oxidase from herbal medicines [14]. These works demonstrate that affinity ultrafiltration coupled with LC-MS technique is applicable to screen and identify active compounds functioning as substrates or inhibitors against the specific target. Compared with traditional screening methods, ultrafiltration offers high recovery yields in screening active compounds from a large number of mixtures, reduction of labor intensity and experimental time. However, low resolution and potential false-positive results are the most notable drawbacks of this technique.

#### 2.1.2. Magnetic Beads or Magnetic Nanoparticles

**Figure 2:**
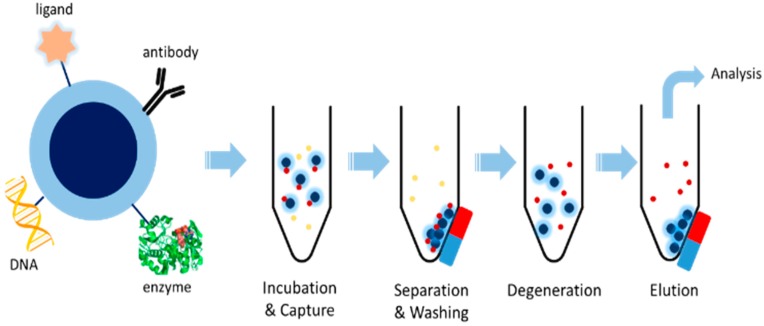
Scheme of magnetic beads based ligand fishing.

On the basis of size scale of particles, magnetic materials can be divided into magnetic beads (or magnetic nanoparticles) and ordinary magnetic materials. Under normal circumstances, the smaller the size of a particle is, the lower a temperature is required for transition from ferromagnetic to superparamagnetism [15]. Due to the smaller particle size of magnetic nanoparticles, they usually exhibit superparamagnetic behavior leading to a more amenable surface structure for modification. Thus magnetic beads are more suitable for immobilizing proteins or enzymes through covalent bonds or adsorption, which can be used later for isolating the known ligand or unknown compound from complex chemical and biological mixtures (Figure 2). Stable immobilization of proteins and easy magnetic isolation are unique advantages of this technique. Because of its accuracy, efficiency and reproducibility, magnetic beads have been widely used in the biomedical field, including protein detection [16], electrochemical biosensing [17], cancer disease treatment [18], drug-protein binding studies [19], etc.

In recent years, significant progress was observed in the adaptation of magnetic beads in bio-active compound discovery from complex natural products. For example, SIRT6 is a histone deacetylase belongs to the Sirtuin family which plays an important role in regulating biological pathways, and was regarded as a potential therapeutic target for metabolic disorders and the prevention of age-associated diseases. Thus Yasuda. et al. established a screening method utilized SIRT6 immobilized magnetic beads for the identification of novel targets of SIRT6 that modulated its biological activity from natural plant extract, and quercetin and vitexin were found have the ability to inhibit SIRT6 activity in vitro, which provides valuable information for understanding their cellular pharmacology [20]. Marszall et al. reported an approach that utilized silica-based magnetic beads that immobilize a heat shock protein 90α for fishing new lead drug candidates and client proteins from complex chemical and biological mixtures, which offers new opportunity for anti-cancer drug discovery [21]. Ji et al. fabricated a novel type of TiO_2_-coated magnetic hollow mesoporous silica spheres (TiO_2_/MHMSS) as a solid phase microextraction (SPME) device to concentrate phosphorylated substrates from EGFR-catalyzed reaction mixtures and MALDI-TOF-MS was employed to structurally characterize these EGFR inhibitors [22].

A number of studies have demonstrated that magnetic bead-based approaches can quickly identify active compounds from diverse kinds of mixtures and its integration with liquid chromatography and mass spectrometry makes it possible to accurately elucidate the structures of these bioactives [23,24]. Compared with traditional methods, magnetic beads exhibit several distinct characteristics. Superparamagnetism of magnetic beads occurs at room temperature with the presence of an external magnetic field, thus there will be no remanence left when the field is removed, which avoids the clustering of magnetic beads and is convenient for recycling. Despite the advantages of magnetic beads described above, some challenges remain to be solved, such as effective desorption of the ligands from the target.

#### 2.1.3. Equilibrium Dialysis

The principle of equilibrium dialysis uses semipermeable dialysis membranes between two small chambers, in which the molecular size decides the diffusion of different molecules, and the target molecule is placed in one of the chambers. After equilibrium, the amount of bound molecule is evaluated by measuring the concentrations of molecule in both chambers. Equilibrium dialysis methods are widely used to examine the binding affinity of ligands to proteins, which is simple and direct, and does not require pretreatment [25].

Hou et al. combined an equilibrium dialysis system with the UPLC-MS/MS approach to analyze and identify the bioactive compounds of Panax Ginseng, which has interactions with the liposome biomembrane, and obtained seven kinds of ginsenosides (Rb1, Rb2, Rc, Rd, Re, Rf, Rg2) that show obvious interactions [26].

#### 2.1.4. Other Materials

Various nanomaterials, such as carbon and TiO_2_ nanotubes have been used as microextraction media for the selective enrichment of specific compounds during the last decade. Our group firstly established lipase-adsorbed halloysite nanotubes (HNTs) for ligand fishing from natural product mixtures. After immobilization by adsorption, the lipase maintains around 80% enzymatic activity. After the approach was validated, this nanotube was applied to screen *Magnoliae cortex* extracts, coupled with HPLC-MS analysis. Four neolignan compounds were identified to be potential inhibitors of lipase, which showed that HNTs can be an available medium for rapid screening of active compounds [27]. Hollow fibers were widely used in the field of sample pretreatment. Some researchers have tried to use them as a new stationary phase. Different from chemical bond processes that may affect the functions of target proteins due to structural alteration, physical adsorption by hollow fiber exhibits easier immobilization process and more diverse selections of stationary material are available. In our previous study, we established a novel approach, named hollow fibers-based affinity selection (HF-AS), for rapid separation and identification of three flavonoids as potential ligands of lipase from extracts of *Lotus leaf*, which suggests an important material basis for the hypolipidemic properties of this herb [28].

Nonspecific binding is an important issue for nanomaterials, including magnetic beads, which can interfere with the identification of real active compounds. As a result, experimental testing using blank material is necessary.

### 2.2. Commonly Used Techniques in On-Line Mode Ligand Fishing

Compared with off-line mode, on-line is regarded as a more attractive manner of ligand fishing. Since the separation and analysis are performed at the same time, more direct results on active compounds would be provided in on-line mode. Meanwhile, real-time analysis makes it possible to realize dynamic monitoring, which is required for obtaining kinetic parameters of interactions.

#### 2.2.1. Biochromatography

Cellular membrane affinity chromatography (CMC) is one of the biological affinity chromatography technologies, in which living cell membranes derived from human or animals are fixed onto a specific support surface to study drug-receptor interactions. CMC takes advantages of the characteristics of classical chromatography, cell biology and receptor kinetics can realized high-throughput and on-line screening from natural products. In addition, CMC has been successfully applied in the bioactive compound discovery. For example, Hou. et al. identified several active compounds that can relax the KCl-treated thoracic artery pre-contracted using vascular smooth muscle cell membrane chromatography coupled with gas chromatography/mass spectrometry from *Radix Angelicae Dahuricae*, *Rhizomza Seu Radix Notopterygii*, *Radix Glehniae*, and *Fructus Cnidii* [29]. Wang et al. developed a new on-line monitoring system for screening epidermal growth factor receptor antagonists from *Radix sophorae flavescentis* through a human epidermal squamous cell membrane chromatography combined with HPLC and MS method [30]. Wu et al. reported a new application of CMC combined with ultra-performance liquid chromatography time-of-flight mass spectrometry for autophagy inducers screening from Raix Polygalae [31].

Compared with the in vitro and in vivo experimental assays, CMC significantly improves screening efficiency with limited labor and time costs. In spite of the advantage of CMC, it also has several drawbacks, such as loss of effective substances, short lifespan of the chromatographic columns, false negatives, and false-positive readings due to nonspecific binding of irrelevant proteins on the membrane. More work needs to be performed to ensure more extensive application of CMC. In general, the CMC technology has good development prospects for screening active compounds from traditional Chinese medicine.

#### 2.2.2. Capillary Electrophoresis

**Figure 3:**
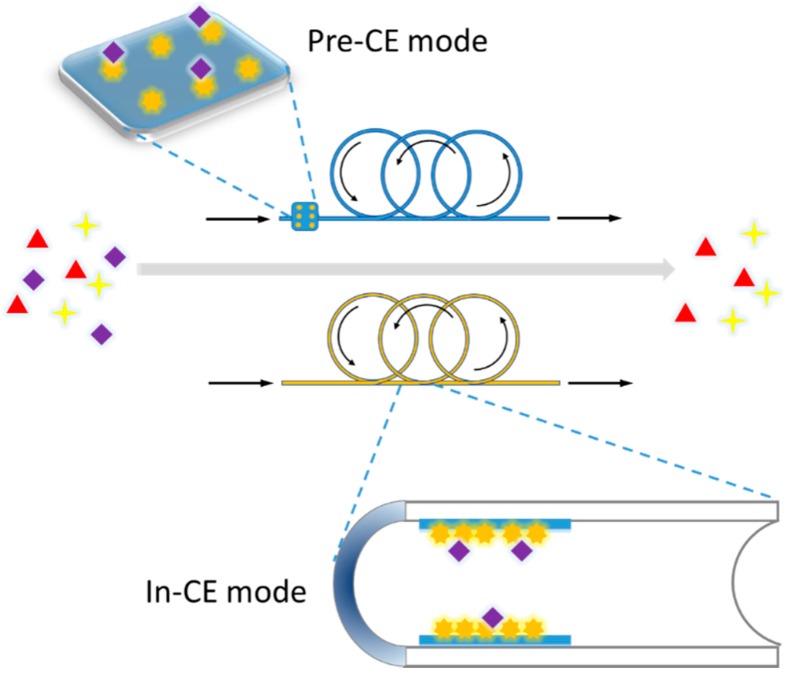
Scheme of capillary electrophoresis based ligand fishing.

Capillary electrophoresis (CE) has been accepted as an efficient separation approach that possesses unique advantages such as high separation efficiency, fast analysis, and minimal sample consumption [32,33]. CE-based enzyme assays account for the majority of its biochemical applications. The enzyme can be bonded on the inner surface of CE or using a bioreactor on the inlet of the CE device, which divides the modes of CE-based enzyme assay into two categories: pre-capillary assays and in-capillary assays (Figure 3). It depends on the location the enzyme reaction happens, in the same capillary or before CE analysis. Because the biochemical assay is integrated into the separation process of CE, the detection avoids interference from the substrate and from the complex sample matrix, leading to high quality assay data from CE-based enzyme assays.

Li et al. reported a method for screening of inhibitors to epidermal growth factor receptor (EGFR) in natural product extracts with CE in conjunction with high performance liquid chromatography-tandem mass spectrometry (HPLC-MS/MS), and 39 natural product extracts derived from TCM were investigated [34]. Ghafourifar et al. directly crosslinked chymotrypsin with glutaraldehyde to produce polymeric particles, which were used inside a fused silica capillary column to make an immobilized enzyme reactor (IMER) that achieved a series of reagent addition and washing steps. A commercial CE instrument is employed to automate the process [35].

#### 2.2.3. Bio-Reactor

Bio-reactors have been an attractive medium for the analysis and screening of bio-active compounds. Among them, enzyme-immobilized microreactors have drawn the most attention and multiple types of surface are applicable to immobilize the enzyme, including glass, agarose, magnetic beads, and many other polymer materials [36]. Immobilized enzyme reactors (IMERs) also emerge as an effective technique in affinity selection for screening potential enzyme inhibitors or activators.

As mentioned above, IMERs are often coupled with CE. A novel CE-based IMER using graphene oxide (GO) as a support was developed by using a simple and reliable immobilization procedure based on layer by layer electrostatic assembly [37]. Similarly, Mohamed et al. used multilayer CE-IMERs for an online assay of the activity and inhibition of *Green tea* extract on the glucose-6-phosphate dehydrogenase (G6PDH), and their results are in good agreement with those obtained from off-line assays [38]. Zhang et al. introduced a new CE method with an IMER for screening α-glucosidase inhibitor from natural products, in which gold nanoparticles are covalently attached to the surface of capillary as a stationary phase [39]. Schejbal et al. created an IMER for the drug metabolizing enzyme, CYP2C9, using magnetic SiMAG-carboxyl microparticles. Their final system integrated the enzyme reaction mixing and incubation, separation, detection and quantification into a single fully automated procedure, realizing an on-line studies of CYP2C9 [40]. In addition to the IMERs, there are other forms of bio-reactors. Mou et al. develop a three-dimensional cell bio-reactor to mimic the cellular structure of real tissues. After coupling with HPLC-MS, this novel system provides a new strategy in affinity screening of lead compounds from herbal medicines [41].

## 3. Applications of Ligand Fishing Approaches in TCM

Many applications have demonstrated that the ligand fishing is a very powerful tool for discovering active compounds from a mixture matrix. As we all know, a TCM is an extremely complex system. Single herbs often contain hundreds of compounds and multiple herbs are combined under the guideline of traditional Chinese medicine theory, which called TCM formula. The chemical complexity of TCM puts forward a real challenge for basic research on active ingredients. The rise of affinity selection techniques provides a novel opportunity to understand the material basis of the pharmacological effects of TCM.

Several groups around the world have reported their efforts in this field. Ping et al. developed a series of methods based on ultrafiltration liquid chromatography/mass spectrometry (UF-LC-MS) for screening high-quality enzyme inhibitors from herbal medicines, [14,42]. For example, they used affinity selection-based two-dimensional chromatography coupled with LC-MS for the online screening of potential xanthine oxidase inhibitors from *Radix Salviae Miltiorrhizae* [43] and antioxidants from the extract of *Ginkgo biloba* (EGB) [44]. Van Breeman et al. developed pulsed ultrafiltration and LC-MS-MS to screen botanical extracts that had affinity with GSH [45]. Moreover, the bioactive phytochemicals of *Angelica sinensis* were investigated guided by the 5-HT7 receptor-binding assay, using human recombinant CHO cell membranes and [3H] LSD [46]. In addition, screening ligands that target to alkylated Keap1 [47], COX-2 [48], quinone reductase-2 [49] by UF-LC-MS was also reported.

In recent years, different ligand fishing approaches were developed to identify active compounds in TCMs against various protein targets (as summarized in Table 1). Liu et al. combined BSA functionalized iron oxide magnetic nanoparticles (BSA-Fe_3_O_4_) with off-line 2D complexation High-speed Countercurrent Chromatography (HSCCC) to separate BSA binders from the ethyl acetate extract of *Fructus polygoni orientalis*, and obtained seven active compounds [50]. Human serum albumin (HSA), the most abundant protein in blood, plays an important role in the transport, distribution and metabolism, thus HSA was widely used as a typical target for ligand fishing. HSA functionalized magnetic nanoparticles (HAS-MNPs) were applied to isolate and identify HSA ligands from *Dioscorea nipponica* extract [51] and *Rheum palmatum* extract [52]. Surface Plasmon Resonance (SPR) is a useful tool for study on intermolecular interactions with dynamic condition and as a real-time monitor. Zhang et al. developed a novel on-line SPR-HPLC-MS/MS system for screening and identification of HSA binders from *Radix Astragali*. In this system, HSA was immobilized on the Au film in the channel by EDC/NHS cross-linking reaction. After incubation and washing step, they obtained twenty compounds, including eleven isoflavonoids and nine astragalosides [53]. Peng et al. established similarly on-line system for fishing HSA binders from *Eucommia ulmoides* bark, and total of 22 compounds from four kinds of iridoids, lignans, flavonoids and phenolic acids were identified as HSA binders by this on-line system [54].

Enzymes occupy the central stage of application of ligand fishing in TCM. Our previous study established α-glucosidase coated magnetic beads for screening enzyme inhibitors from *Morus alba* [55]. α-Amylase plays an important role in the digestion of carbohydrates, which means finding inhibitors of α-amylase is a potential strategy for glycemic control in type-2 diabetes. Li et al. firstly established the protein immobilized magnetic fishing method combined with HPLC to screen α-amylase inhibitors from *G. xanthochymus*, which showed antidiabetic activities in previous studies [56]. Recent studies suggested that curcuminoids from *Turmeric* showed significant inhibition of COX-1. Zhang et al. sequentially developed screening assays based on COX-1 and COX-2 functionalized magnetic nanoparticles for ligand fishing. By Fe_3_O_4_@SiO_2_-COX-1 coupling with mass spectrometry, they isolated four curcuminoids from *Turmeric* extraction as COX-1 potential inhibitors [57], and Fe_3_O_4_@SiO_2_-COX-2 combination with HPLC-DAD-MSn was applied to screen and identify COX-2 inhibitors from *Green tea* [58]. Acetylcholinesterase (AChE) inhibitors are one of the available therapies for Alzheimer’s disease, and many known inhibitors were derived from plant extracts. Vanzolini et al. chose AChE as the target and coated it to magnetic beads, which was used as the bioreactor for the screening of *Melodinus fusiformis* extracts. Meanwhile, they also used non-linear zonal bioaffinity chromatography with AChE-ICER to assess the inhibitors activity [59]. Tyrosinase is a multifunctional type-3 copper-containing metalloenzyme related to the reactions of the melanogenesis process, Liu et al. applied tyrosinase immobilized magnetic nanoparticles (MNPs) coupled with HPLC-DAD-MS/MS and reverse ultrafiltration-HPLC, respectively, for screening tyrosinase binders from *Glycyrrhiza uralensis root*. Eleven potential inhibiters were identified, and may have effect on various dermatological disorders [60]. GSK-3β, a multifunctional serine/threonine kinase considered as potential target for diabetes mellitus and neurodegenerative diseases, was immobilized on magnetic beads for screening inhibitors from extracts of 15 traditional Chinese medicines [61].

In addition to enzymes, there are other possible targets that used for ligand fishing from TCM. Xu et al. focused their attention on screening triplex DNA binders from natural plant extracts, and for this purpose, they used agarose beads immobilized triplex DNA coupled HPLC-ESI-MS for ligand fishing. Two compounds were fished out from the extracts of *Phellodendron chinense Schneid cortexes* by this assay, extending the application of ligand fishing [63]. G protein coupled receptors (GPCRs) are important in the treatment of many diseases. Liu et al. developed an immobilized β2-adrenergic receptor (β2-AR) CE to determine the interactions between a set of natural extracts of *Radix Paeoniae Rubra* (NERPR) and β2-AR. The inner capillary surface is chemically bonded with stable β2-AR coating via microwave-assisted technical synthesis. The results confirmed that this is a potential way to select lead compounds from natural chemicals [64].

## 4. Future Perspectives of Ligand Fishing for TCM

### 4.1. Screening Active Compounds Based on the Multi-Target

As shown in the previous section, most ligand fishing studies concentrate on a single target. However, it is well known that TCMs exert their beneficial effect through multiple components, multiple targets and multiple pathways. Thus it is important to expand current affinity selection techniques to fulfill the screening on multi-target or multi-channel. Different kinds of targets can be immobilized on the surface of magnetic beads or nanomaterials, so that they can be applied into screening for more than one active compound at one time.

Zhao et al. realized on-line dual-targets screening from complex matrix by immobilizing two kinds of G-protein-coupled receptors, α1-adrenoceptor (α1A-AR) and β2-adrenoceptor (β2-AR), on the surface of macroporous silica gel. The extracts from *Salvia miltiorrhiza* and *Coptis chinensis* are analyzed by this biochromatography column, leading to three compounds active for both receptors [65]. Our group developed a multi-target screen system using multiple-immobilized magnetic beads coupled with HPLC/MS. Maltase, invertase, lipase were immobilized on the magnetic beads separately, and the beads were placed into three connected chambers. This system was then applied for screening active compounds from a Chinese medicine called “*Tang-Zhi-Qing*”, a modern formula consisted of *Mulberry leaves*, *Lotus leaf*, and *Salvia miltiorrhiza*, has effects on decreasing sugar and regulating lipid, and five compounds were identified and their activities were validated [67].

### 4.2. Screening Active Compounds Based on Microfluidic

Microfluidic reaction devices that are manufactured by microfabrication techniques, or by assembly and modification of polymer layers [68], constitute reaction system with small dimensions, large surface to volume ratios and well defined reaction times. In the field of microfluidic lab-on-a-chip systems, the centrifugal microfluidic platform has emerged as an advanced technology for biological analysis [69]. The related applications include clinical chemistry, immunodiagnostics and protein analysis, cell handling, molecular diagnostics, as well as food, water, and soil analysis [70], and most commercial products are used for biomarker identification in the clinic. Affinity selection is also possible to be re-designed for microfluidic assay. Multiple channels of the system allow the combination of several functional units, e.g., screening for multiple targets. Some affinity separation techniques reviewed above are ready to be integrated with microfluidic system, such as magnetic beads. Though there are not yet reports on such system designed for active compounds screening, it is possible in theory due to the common procedures of incubation, wash, and elution that already existed [71]. Lab-on-a-chip or lab-on-a-CD is another feasible strategy for high-throughput screening, which would contribute significantly to chemical and pharmacological research of TCM.

## 5. Conclusions

In this review, we focused on the ligand fishing technique, an emerging field in affinity separation techniques. These techniques hold great promise in screening bio-active compounds from natural mixtures with high efficiency, a high hit rate, stabilization and reliability. Its applicability in TCM has been confirmed on several protein targets, especially enzymes. However, limited to the unknown relationship between the constituents of complex mixture and their targets, ligand fishing can be applied to novel targets including enzymes, nuclear receptors that tightly related to the pathophysiological process. More efforts need to be spent on designing ligand fishing systems with multiple-targets or multiple-channels screening capability, which may accelerate our understanding of the mechanism of action of complex natural products.

## Figures and Tables

**Table 1:** Applications on TCM by various ligand fishing strategies.

No.	Herb	Target	Method	Active Compounds	Reference
1	*Eugenia catharinae*	α‑Glucosidase	Enzyme coated magnetic beads	5-(2-Oxopentyl)resorcinol 4-*O*-β-d-glucopyranoside, 5-propylresorcinol 4-*O*-β-d-glucopyranoside, 5-pentylresorcinol 4-*O*-[α-d-apiofuranosyl-(1→6)]-β-d-glucopyranoside, 5-pentylresorcinol 4-*O*-β-d-glucopyranoside, 4-hydroxy-3-*O*-methyl-5-pentylresorcinol 1-*O*-β-d-glucopyranoside, 3-*O*-methyl-5-pentylresorcinol 1-*O*-[β-d-glucopyranosyl-(1→6)]-β-d-glucopyranoside	[62]
2	*Morus alba*	α-Glucosidase	Enzyme coated magnetic beads	Isoquercitrin, astragalin	[55]
3	*Garcinia xanthochymus*	α-amylase	Enzyme coated magnetic nanoparticles	GB2a glucoside, GB2a, fukugetin	[56]
4	*Turmeric*	Cyclooxygenase-1 (COX-1)	Enzyme coated magnetic nanoparticles	Curcumin, demethoxycurcumin, bisdemethoxycurcumin, 1-(4-hydroxy-3,5-dimethoxyphenyl)-7-(4-hydroxy-3-methoxyphenyl)-(1*E*,6*E*)-1,6-heptadiene-3,5-dione	[57]
5	*Green tea*	Cyclooxygenase-2 (COX-2)	Enzyme coated magnetic nanoparticles	(−)-Epigallocatechin-3-(3”-*O*-methyl)-gallate, (−)-epicatechin-3-(3”-*O*-methyl)-gallate	[58]
6	*Radix Salviae Miltiorrhizae*	Xanthine oxidase	Affinity selection-based 2D chromatography coupled with LC-MS	Salvianolic acid C, Salvianolic acid A	[43]
7	*Melodinustenuicaudatus*	Acetylcholinesterase (AChE)	ICERsand enzymes coated to magnetic beads	An active compound from fraction 1	[59]
8	*Euonymus fortunei and G. xanthochymus*	GSK-3β	Magnetic beads	Fukugetin	[61]
9	*Dioscorea nipponica*	HSA	HSAfunctionalized magnetic nanoparticles	Dioscin, gracillin, pseudo-protodioscin	[51]
10	*Radix Astragali*	HSA	SPR-HPLC-MS/MS	11 Isofalvonoids and 9 astragalosides	[53]
11	*Eucommia ulmoides*	HSA	SPR-HPLC-MS/MS	4 Iridoids, 11 lignans, 3 flavonoids, 4 phenolic acids	[54]
12	*Fructus polygoni orientalis*	BSA	off-line2D complexation HSCCC	3,5,7-Trihydroxychromone, taxifolin, *N*-*cis*-paprazine, *N*-*cis*-feruloyltyramine, *N*-*trans*-paprazine, *N*-*trans*-feruloyltyramine, an unidentified compound	[50]
13	*Phellodendronchinense Schneid cortexes*	Triplex DNA	triplex DNA immobilized agarose beads	Berberine, palmatine	[63]
14	*Radix Paeoniae Rubra*	β_2_-AR	β_2_-AR immobilized CE	Extract of B18-19-②	[64]
15	*Miltiorrhiza and Coptis chinensis*	alpha1-adrenoceptor (α_1A_-AR) and beta2-adrenoceptor (β_2_-AR)	immobilized on the surface of macroporous silica gel	Berberine, palmatine, jatrorrhizine	[65]
16	*Fenugreek seed extract*	SIRT6	SIRT6 coated magnetic beads	Quercetin, vitexin	[20]
17	*Trigonella foenum-graecum*	SIRT6	SIRT6 coated magnetic beads	Orientin and 17 other compounds	[66]
18	*Glycyrrhiza uralensis root*	Tyrosinase	Enzyme immobilized magnetic fishing coupled with HPLC-DAD-MS/MS	Liquiritinapioside, neolicuroside, liquiritigenin, licorice saponin G2, chrysoeriol, dihydrodaidzein, formononetin, glycyrrhisoflavanone, glycyrrhizic acid, licoarylcoumarin, pratensein	[60]
19	*Mulberry leaves*	Tyrosinase	Ultrafiltration LC-MS	Quercetin-3-*O*-(6-*O*-malonyl)-β-d-glucopyranoside and kaempferol-3-*O*-(6-*O*-malonyl)-β-d-glucopyranoside	[13]
20	*Lotus leaf*	Lipase	Hollow fibers	Quercetin-3-*O*-β-d-arabinopyranosyl-(1→2)-β-d-galactopyranoside, quercetin-3-*O*-β-d-glucuronide, kaempferol-3-*O*-β-d-glucuronide	[28]
21	*Magnoliae cortex*	Lipase	Lipase-adsorbed nanotube combined with HPLC-MS analysis	Magnotriol A, magnaldehyde B	[27]
22	*Tang-Zhi-Qing*	Maltase, invertase, lipase	Magnetic beads based multi-target affinity selection-mass spectrometry	2,3,4,6-Tetra-*O*-galloyl-d-glucose, 1,2,3,4-tetra-*O*-galloyl-d-glucose, 1,2,3,4,6-penta-*O*-galloyl-d-glucose, quercetin-3-*O*-β-d-glucuronide, quercetin-3-*O*-β-d-glucoside	[67]
23	*Radix Angelicae Dahuricae, Rhizomza Seu Radix Notopterygii, Radix Glehniae,* and *Fructus Cnidii*	L-calcium channel receptors	vascular smooth muscle cell membrane affinity chromatography	Imperatorin and osthole	[29]
24	*Radix sophorae flavescentis*	Epidermal growth factor receptor (EGFR)	A(431)/cell membrane chromatography-HPLC/MS	Oxymatrine and matrine	[30]
25	*Radix Polygalae*	Neuronal cells	PC12 cell membrane chromatography-UHPLC-(Q)TOF-MS	Onjisaponin B	[31]
